# Actical Accelerometers as a Clinical Tool for the Monitoring of Sleeping and Resting Periods in Individual Dogs

**DOI:** 10.3390/ani15172571

**Published:** 2025-09-01

**Authors:** Simone Straube-Koegler, Britta Dobenecker, Susanne Lauer, Franziska Wielaender, Yury Zablotski, Andrea Fischer

**Affiliations:** 1 LMU Small Animal Clinic, Centre for Clinical Veterinary Medicine, Ludwig-Maximilians-Universitaet Muenchen, 80539 Munich, Germany; 2 Chair of Animal Nutrition and Dietetics, Ludwig-Maximilians-Universitaet Muenchen, 80539 Munich, Germany; 3 Small Animal Clinic Oberhaching, 82041 Oberhaching, Germany

**Keywords:** rest–activity patterns, canine health, behavior, actigraphy, companion animals, home-based monitoring, neurology

## Abstract

Various accelerometers offer to monitor sleep efficiency in dogs, but the underlying proprietary algorithms are not usually revealed. The aim of this pilot study was to achieve insights on the performance of an omnidirectional accelerometer to document sleeping and resting periods in dogs with a simple binary cut-off (0 cpm; ≥1 cpm) and the interference of movements. All data were acquired with a neck collar-mounted accelerometer (Actical^®^; Philips Respironics Inc., Murrysville, PA, USA) that provides access to raw data. Ten privately owned dogs (6 dogs with and 4 dogs without movements during sleep) were monitored shortly before, during and after their sleeping and resting periods. Experienced observers documented any visible movement and the corresponding behavioral state continuously for each minute as apparent sleep (eyes closed, regular breathing pattern), rest (eyes open), awake and alert. Total observation time was 2633 min, and 32 sleeping and resting periods were monitored. Zero accelerometer counts per minute documented the combined sleeping and resting periods with a sensitivity of 94.0% and specificity of 96.1% but could not differentiate between apparent sleeping and resting with eyes closed or open. Mild movements during sleep did not interfere with the recordings. The pilot data obtained in this study supports further investigation of this approach to document sleeping and resting periods over prolonged periods in individual dogs.

## Simple Summary

The monitoring of sleeping and resting periods is underutilized in clinics and could achieve insights into the well-being and health in dogs. Accelerometers usually inform sleep efficiency in dogs, but the underlying proprietary algorithms are not disclosed. There is a need for a simple and reliable tool that can be used in clinics and research and provides access to raw data. In this pilot study, ten privately owned large-breed dogs, with or without a history of movements during sleep, were fitted with a collar-mounted Actical^®^ accelerometer and monitored in detail for each minute shortly before, during, and after their individual sleeping (eyes closed, regular breathing pattern) and resting periods (eyes open). Zero accelerometer counts per minute indicated sleeping and resting with a high sensitivity and specificity, while the accelerometer effectively captured moderate and strong movements of the head and neck. The data support further investigation of this approach in long-term studies and larger cohorts. The device should be considered a clinical tool for monitoring sleeping and resting periods in individual dogs.

## 1. Introduction

Sleep serves as a pivotal indicator of health and disease states [1,2,3,4]. There is a need to establish universally recognized gold standards for the objective and longitudinal assessment of sleeping and resting phases in dogs. Polysomnography (PSG) can document sleep stages and detect sleep disorders by recording physiological signals during sleep, such as selected electroencephalographic traces in association with ocular movements, muscle activity, heart and respiratory rate, but recordings in dogs are often short-lived and restricted to a few hours during afternoon naps [5,6,7,8,9,10,11]. Furthermore, polysomnographic examinations are time-consuming, and their interpretation requires experience [12].

Actigraphy is commonly used as an adjunct diagnostic and objective method to PSG in human medicine. Actigraphy monitors movements with a wearable device and estimates the duration of sleep/rest periods based on motion data. While PSG provides detailed and accurate information about sleep onset and sleep stages, actigraphy offers a less precise but practical solution for the monitoring of activity–rest patterns during the night and in naturalistic settings. Actigraphy assists with the monitoring of sleep and documentation of wake phases during the night. This is especially helpful in patients who present for investigation of sleep disorders or for monitoring the response to therapy [13,14,15,16,17]. In this context, actigraphy is considered an objective complement to sleep diaries, which can only document perceived sleep phases from the patient’s perspective [18,19].

Various accelerometers are in use in veterinary medicine. In dogs, accelerometers are mainly used to assess different activity levels, e.g., whether the dog is resting, walking, or running. Specific thresholds (e.g., counts per minute) correlate with specific activity levels [4,20,21,22,23]. The Actical^®^ accelerometer has previously been validated for monitoring spontaneous at-home activity in dogs [24,25,26] and recording the physical activity of healthy dogs [21,27,28,29,30], and deviations from normal patterns in dogs with chronic pain from osteoarthritis or cognitive dysfunction [31,32,33,34,35,36,37,38]. The Actical^®^ accelerometers serve as a benchmark and pioneer for all subsequent devices [32,39,40]. Actical^®^ accelerometers have the advantage of allowing the collection of raw, unfiltered accelerometer data for prolonged periods, up to several weeks or months. Furthermore, they collect and store data independently from internet access locally on the device. Thus, the risk of loss of data is minimal. 

Various marketed accelerometers offer to monitor sleep efficiency. At present, there is no consensus on which accelerometer should be used by default for the documentation of sleep and rest periods in dogs. Some research groups aimed to document the resting phases of dogs based on their head position and applied individual algorithms that were adapted to the device [41,42]. Devices from different manufacturers use proprietary algorithms [43,44]. Therefore, it is problematic to apply the same thresholds across different devices. More recently, Actical^®^ accelerometers were shown to successfully document fluctuation in circadian sleep–wake cycles in privately owned dogs in their home environment [36,38].

The aim of this study was to investigate in a pilot study whether an Actical^®^ accelerometer could reliably document sleep and rest periods in dogs in their home environment, using a simple and transparent approach that has previously not been applied for this purpose. We hypothesized that 0 counts per minute (cpm) would indicate sleeping or quiet resting, and counts ≥ 1 cpm would indicate an awake and alert behavioral state. A secondary aim was to assess the impact of minor movements during sleeping and resting on the recordings.

## 2. Materials and Methods

The animal studies were approved by the ethics committee of the faculty of veterinary medicine, LMU Munich (protocol number 133-13-07-2018). The study was conducted in accordance with the local legislation and institutional requirements. Written informed consent was obtained from the owners for the participation of their animals in this study.

### 2.1. Study Design

The study was designed as a pilot study to obtain data on the performance of an accelerometer for the documentation of sleeping and resting periods in dogs with a simple binary cut-off, and to assess the interference of movements.

**Figure 1:**
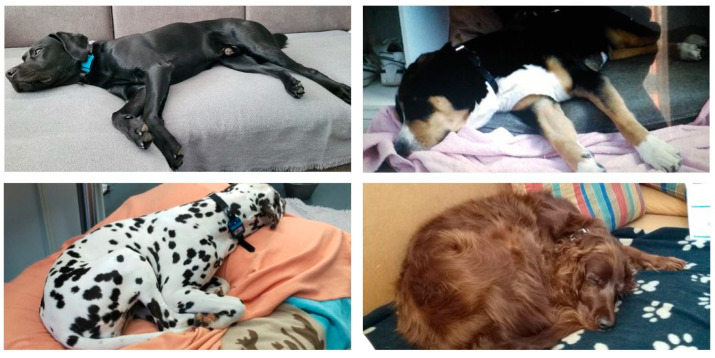
Dogs monitored during their usual sleeping and resting periods.

Ten privately owned dogs (6 dogs with and 4 dogs without movements reminiscent of rapid eye movement (REM) sleep during sleep) were fitted with collar-mounted accelerometers (Actical^®^; Philips Respironics Inc., Murrysville, PA, USA) with epochs set at one minute. Each dog was observed by a single observer in its home environment shortly before, during, and after its usual sleeping and resting time in the afternoon or evening (Figure 1).

The observers received instructions to watch the dog continuously from the beginning to the end of its usual sleeping and resting time, to provide written documentation on the behavioral state of the dog, and to document any visible movements of the head, facial muscles, limbs, paws, neck, or trunk in detail for each minute. For data analysis, we compared the documentation with the corresponding accelerometer counts and evaluated the performance of the accelerometer with a simple binary cut-off, e.g., whether a count of 0 cpm vs. ≥1 cpm could differentiate between sleep or quiet rest and an awake and alert behavioral state.

### 2.2. Animals

Ten client-owned pet dogs (9 female, 1 male; mean age: 5.4 years, median age: 3.3 years) of eight different breeds participated in the study: three Labrador Retrievers, a Weimaraner, a Rhodesian Ridgeback, a German Shepherd, an Irish Setter, a Bernese Mountain Dog, a Dalmatian, and a mixed breed dog. All dogs were healthy based on physical examination and their owners’ reports. Four pet owners reported that their dog frequently would show movements during sleep, e.g., twitching of the nose, lips, and paws when sleeping, and two more dogs showed these movements in the study. These movements were reminiscent of REM sleep even though rapid eye movements were typically not picked up by the observers and accelerometers as well (Table 1).

### 2.3. Accelerometer

**Figure 2:**
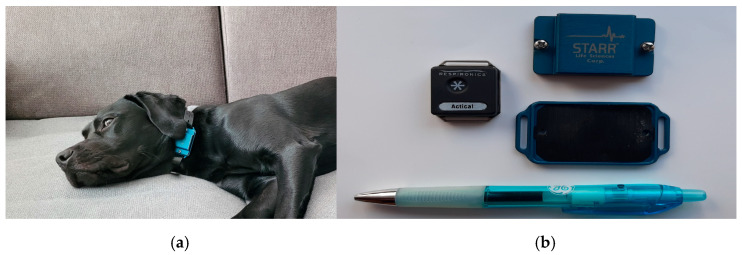
(**a**) Labrador retriever with an Actical^®^ accelerometer. The dog wears the accelerometer on a neck collar while monitored during sleeping and resting periods at home. (**b**) The Actical^®^ accelerometer and the metal protective cover with upper and lower parts are shown on the right (Philips Respironics Inc., Starr Life Sciences Corp.). A standard ballpoint pen is shown for size comparison.

A collar-mounted accelerometer (Actical^®^, Philips Respironics Inc.) was used in all dogs (Figure 2). The accelerometer was equipped with a new battery, pre-programmed and activated prior to each recording. Epoch length was set at one minute [24]. The time and date of the accelerometer were synchronized with the computer and the observer’s watch. The accelerometer was placed within a metal protective case and threaded onto the provided collar (Starr Life Sciences Corp., Oakmont, PA, USA). The collar was placed on the neck of the dog with the accelerometer positioned at the ventral region of the neck [26]. There was a two-finger-width distance between the collar and the dog’s neck.

### 2.4. Observation

The observation and recording periods were adapted to the daily routine of each dog. After the fitting, the dog was taken to a familiar room, e.g., the living room at home or the owner’s office at the clinic, where it usually spent its resting time during the afternoon or early evening. The observers of the dogs were four veterinarians and two veterinary technicians. Each dog was continuously observed during several sleeping and resting periods by a veterinarian (pet owner), veterinary technician (pet owner), or study director, who was already familiar with the dogs. Observers were in the same room with the dog and were instructed to maintain an unrestricted view of the entire dog and especially the head without interfering with the dog’s ability to choose its usual sleeping place but no fixed distance was defined. Written documentation was obtained for each minute corresponding to the one-minute epoch of the accelerometer. The observers were instructed to document whether the dog appeared to sleep (lies motionless with closed eyes, regular breathing pattern), rested quietly (lies motionless with eyes open), or was awake and alert. Additionally, any visible movements or changes in body position were documented. The observation and recording period continued until the dog woke up and obviously did not want to rest anymore, e.g., started to play and interact with its environment.

### 2.5. Review

The Actical^®^ data was read out and exported to Microsoft Excel^®^ using the proprietary software (Philips Respironics Inc., PA, USA). We reviewed the Actical^®^ counts for each minute (cpm) and applied two Actical^®^ categories using a simple binary cut-off (0 cpm; ≥1 cpm). We reviewed the written documentation of the observers (minutes of apparent sleep, rest, awake and alert behavioral state; any visible movements), compared written documentation and Actical^®^ data for each observer (quality check for missing data), and applied movement categories (M0, M1, M2, M3, M4) based on the intensity of the observed movements (Table 2). The study design did not permit formal statistical analysis for inter-observer reliability, or to validate subjective behavioral classifications.

Agreement upon the behavioral state of the dogs between the written documentation and the two Actical^®^ categories (0 cpm, ≥1 cpm) was evaluated. Furthermore, the ability of the Actical^®^ cut-off to document movements of different intensities (movement categories M0–M4) was assessed. Sensitivity and specificity, including 95% confidence intervals of the Actical^®^ cut-off to document sleeping and resting, were calculated. The quality of the cut-off to discriminate between behavioral states was further evaluated with the McNemar test and the area under the curve (AUC) derived from receiver operating characteristics (ROC). Positive and negative likelihood ratios (prevalence independent parameters), positive predictive value (PPV), and negative predictive value (NPV), which apply to the use of the cut-off in a similar monitoring situation (prevalence dependent parameters), were calculated. Positive and negative likelihood ratios were calculated as sensitivity/(1-specificity) and (1-sensitivity)/specificity, respectively [45,46,47]. All calculations and statistical analyses were conducted using R statistical software (R version 4.2.1; 23 June 2022) and Microsoft Excel^®^ (Microsoft Office 2016).

## 3. Results

In total, 32 sleep and rest periods (median duration 94 min) were monitored in the 10 dogs (Table 1). The total observation time was 2633 min. Written documentation was missing for 16 min, resulting in 2617 min (43.6 h) of valid observations: 2363 min sleep and rest, 254 min awake and alert behavioral state. The mean observation time for each dog was 261.5 min (median 229, range 173–468 min).

### 3.1. Documentation of Movements

**Figure 3:**
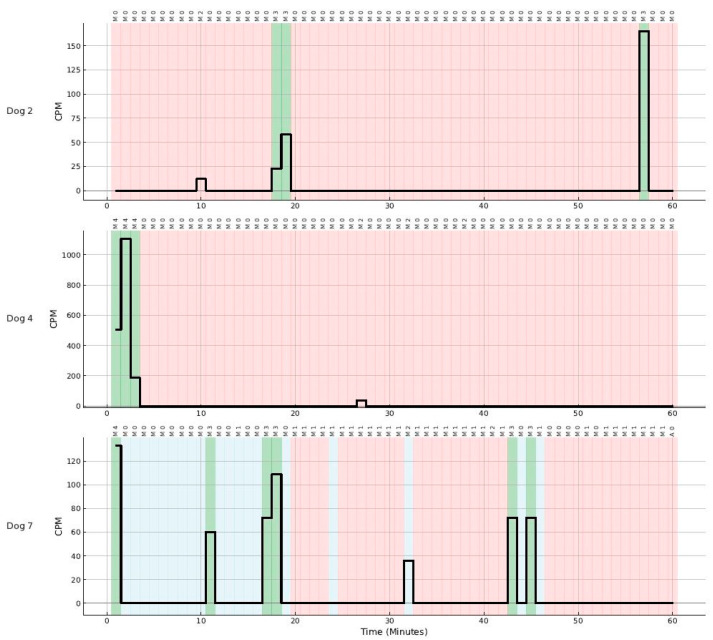
Time-synchronized accelerometer data and corresponding observer-recorded behavior for three dogs. Each panel represents a 60-min observation period for one dog. Background colours indicate behavioral state: red—apparent sleep (eyes closed, regular breathing); blue—rest with eyes open; green—awake and alert. The black trace shows accelerometer output in counts per minute (CPM). The upper row above each panel indicates observed movement intensity (M0—no movement; M1—mild; M2—moderate; M3—strong; M4—purposeful activity).

The observers described 1762 min without any visible movements (M0) and 855 min with movements of different intensities (M1–M4). Mild and moderate movements were sometimes observed, while the dog was sleeping or resting. The collar-mounted accelerometer recorded mild movements only rarely (2.9%; M1) and recorded only 50.8% of the moderate movements (M2). Strong movements were always documented by the accelerometer (100%; M3). Movements during an awake and alert behavioral state and purposeful activities were always documented by the accelerometer (100%; M4). Figure 3 illustrates time-synchronized accelerometer output and observer scoring of behavioral state and movement intensity (Figure 3).

- M0: There were 1762 min in which the dog did not show any movements. These were 1628 min of apparent sleep (eyes closed), 124 min of rest (eyes open)*,* and 10 min when the dogs were awake and alert and interacted with the environment but were sitting or lying motionless, e.g., waiting for a command. The Actical^®^ accelerometer documented 99.4% (1742/1752 min) of the sleep and rest minutes correctly as 0 cpm and in line with the written observations.
- M1: Mild movements occurred for 375 min. Mild movements occurred in this experiment during sleeping or resting. The written documentation showed that mild movements consisted frequently of paw and nose twitching. The Actical^®^ accelerometer failed to document these movements in 97.1% of the evaluated time (364/375 min; 0 cpm). Mild movements, which were not detected by the Actical^®^, occurred frequently when the dogs appeared to sleep and less commonly when the dogs were resting quietly.
- M2: Moderate movements occurred for 236 min. Moderate movements occurred during sleep or rest. These movements occurred only infrequently during sleep, and more frequently when the dogs were resting quietly. The Actical^®^ documented movements in 50.8% of the evaluated minutes (120/236 min, ≥1 cpm) and failed to detect 49.2% (116/236 min; 0 cpm) of the minutes. Overall, moderate movements were recorded when the dog lifted the head and neck but were not recorded when only the limbs moved (e.g., stretched), and the dogs remained in their lying position without any movement of the head or neck. Therefore, more movement and activity of the head and neck took place, and the sleep and rest periods were interrupted when the Actical^®^ recorded ≥1 cpm. The sleep and rest periods were not interrupted and only limb stretching took place if the Actical^®^ recorded 0 cpm.
- M3: Strong movements occurred for 49 min and always during an awake and alert behavioral state. These movements were always recorded by the Actical^®^ accelerometer (≥1 cpm).
- M4: Pronounced movements and purposeful activities in association with an awake and alert behavioral state occurred for 195 min. Pronounced movements occurred shortly before the sleeping and resting periods started or when the dog woke up. These movements were always detected by the Actical^®^ accelerometer (≥1 cpm).

Table 3 shows the range of the measured Actical^®^ counts (cpm) for each movement category (M0–M4) and the corresponding behavioral state.

### 3.2. Documentation of Sleeping and Resting Periods

**Figure 4:**
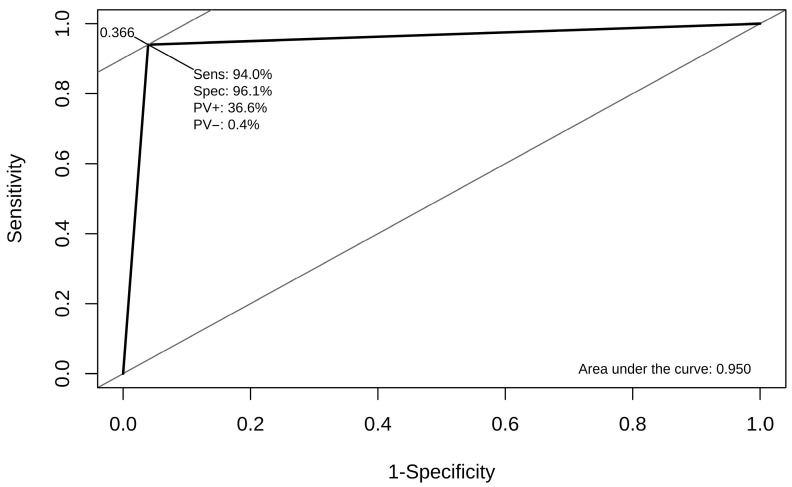
Performance of the cut-off. The Actical^®^ cut-off (0 cpm vs. ≥1 cpm) appeared well suited to document the combined sleeping and resting periods of dogs. The ROC curve indicated 94.0% sensitivity and 96.1% specificity. The area under the curve was 0.950.

The Actical^®^ accelerometer classified 94.0% of the observed sleep and rest minutes correctly as sleeping or resting (0 cpm) when a binary cut-off was used with 0 cpm indicative of sleep or rest and ≥1 cpm indicative of an awake and alert behavioral state Analysis of the ROC curve indicated that the cut-off is well suited to document sleep–rest periods in dogs (Figure 4). Sleep and rest periods were recorded with a sensitivity of 94.0% and a specificity of 96.1%, and the AUC was 0.950.

Further comparison of the sleeping and resting minutes indicated that the Actical^®^ cut-off could not differentiate between sleeping and resting (specificity 0.273; AUC 0.627). The overall performance of the Actical^®^ accelerometer is summarized in Table 4.

## 4. Discussion

The aim of the study was to document sleeping and resting periods in dogs in detail for every minute and to evaluate the performance of the Actical^®^ accelerometer for the documentation of sleeping and resting periods in individual dogs. The accelerometer can detect quiescence, but it cannot detect sleep architecture, and also not when the dogs are awake but motionless.

The study was designed as a pilot study with a special focus on the possible interference of movements during sleep with the accelerometer recordings. Therefore the study population included six dogs with movements during sleep which were reminiscent of REM sleep and four dogs without those movements. The results showed that a simple cut-off criterion (0 cpm; ≥1 cpm) for each recorded minute, which could be used in daily veterinary practice, detected and documented each minute of sleeping and resting with a sensitivity of 94.0% and a specificity of 96.1%. Small movements during sleeping or resting, including movements reminiscent of REM sleep, did not interfere with the recordings when the head, neck, and trunk did not move. Therefore, the Actical^®^ accelerometer appeared well suited to monitor and document combined sleeping and resting periods in dogs in their home environment. Further validation of this concept in larger study cohorts with long-term monitoring and simultaneous video documentation is warranted.

The monitoring of sleeping and resting periods is underutilized in veterinary medicine [48,49,50,51,52]. There is an increasing awareness that sleeping and resting periods are as important for the well-being and health of dogs as they are in humans, and there is a need for objective monitoring tools. The importance of sleep in neurobehavioral functions, including emotional regulation and memory consolidation, immune control, and promotion of recovery, is well known in human medicine [53,54]. Furthermore, there is a strong research interest in the interaction between sleep and daytime activity, and also between sleep and epilepsy in dogs [9,55]. In summary, sleep is essential for health in dogs as in humans, and sleep deprivation can have serious negative consequences [1,2,3]. Accelerometers offer a wide range of clinical applications in dogs. Accelerometers could be useful to document aimless wandering and restlessness during night in dogs with dementia, i.e., from altered sleep–wake cycles in canine cognitive dysfunction syndrome, or the negative impact of chronic pain or pruritus on sleep and rest during night [33,36,38,50,51]. Other potential applications are altered sleep and rest behavior in brachycephalic dogs with obstructed airways, in dogs with REM sleep behavior disorder or canine idiopathic epilepsy [55,56,57,58]. Furthermore, accelerometers also offer a practical method to assess rest patterns as indicators of overall welfare in shelter dogs [59].

Actical^®^ accelerometry has been validated previously to document fitness and activity in dogs, and it is currently considered the gold standard of accelerometry in dogs [21,24,25,26,32,40,60]. In recent years, several other accelerometer studies aimed to record various behavioral states of dogs, e.g., running, walking, sleeping, and resting [21,22,61], using device-specific algorithms to calculate the optimal cut-off values [41,42,43,44]. The use of proprietary technology makes it impossible to implement these algorithms across devices. Furthermore, the knowledge of the accuracy of accelerometers for monitoring sleeping and resting periods in dogs is still limited [62]. Many accelerometers report on sleep efficiency without revealing underlying algorithms and without differentiating between sleeping and resting. In contrast, actigraphy has gained its place in sleep studies in human medicine. It offers an easy, non-invasive, and objective tool that can supplement or even serve as a substitute for lengthy written documentation of perceived sleep and wake time [13,15,16,17,18].

A concern was whether the accelerometer would record small movements occurring during sleep and thus falsely assign an awake and alert behavioral state. Sleep movements, such as paw twitching, occur frequently during REM sleep. Reviewing the observed movements and the corresponding accelerometer counts showed that the accelerometer did not record mild movements during sleep, which were reminiscent of REM sleep movements, nor did it record moderate movements, which occurred during resting, e.g., when the dog extended or stretched the limbs without any movement of the head, neck or trunk. On the other hand, moderate movements of the head and neck, e.g., when the dog lifted the head and looked around, and any unequivocal interruption of the sleeping and resting period, e.g., when the dog stood up, turned around, or relocated to a different resting place, were consistently associated with ≥1 counts per minute and thus documented as interruption of the sleeping and resting period. Occasionally, more pronounced limb movements may occur, and individual dogs could even show signs of a REM sleep behavior disorder, which is characterized by violent movements of limbs and bodies and howling in association with REM sleep [56,63]. It is a limitation that no dogs with a known REM sleep disorder participated in this study. In summary, the data proved that Actical^®^ accelerometers could be used to document sleeping and resting periods and their interruption in detail with a sensitivity of 94.0% and specificity of 96.1% in individual dogs. The 0 cpm vs. ≥1 cpm cut-off achieved excellent positive and negative predictive values for the minute-by-minute documentation of sleeping and resting periods. Yet, one should consider that the PPV and NPV are strongly influenced by the prevalence of the specific event, and more realistic data could be obtained when the performance of the cut-off is studied in different situations. However, likelihood ratios are not influenced by prevalence and support the validity of our data [45,46,47].

As expected, the results demonstrated that the accelerometer was only able to detect movements and could not distinguish between sleeping and resting. Also, the device failed to document an awake and alert state when the dogs were sitting motionless for a few minutes and waited, e.g., for a command from the owner or observer, for instance, to catch a ball or to approach the feeding bowl. In these situations, the dogs were sitting or lying without any visible movements, but the dogs were obviously in an awake and alert behavioral state and attempted to interact with the environment. The drawbacks of actigraphy are, therefore, the limited specificity to assess the behavioral state when the dogs are lying motionless. This mirrors the situation in human medicine, where various commercially available algorithms are in use for assessing sleep parameters (e.g., sleep duration and sleep efficiency) [64]. The Sade algorithm has been validated in children and adolescents against the gold standard PSG, and it is the most commonly used algorithm [65,66]. One of the major restrictions of this and other algorithms is the poor accuracy in detecting wake after sleep onset when subjects may be lying awake, but motionless, leading to an overestimation of sleep [15,67,68]. This parallels, to some degree, the observations with the Actical^®^ accelerometer in dogs in this study. The Actical^®^ accelerometer was unable to differentiate between apparent sleep with closed eyes and a regular breathing pattern and rest with eyes open. In this context, the specificity decreased to 27.3%. The specificity could have been even lower, considering that dogs could have rested with closed eyes. These results are expected because both behavioral states, sleeping and resting, are usually not associated with movements, and only PSG can differentiate between the awake resting behavioral state and sleep [5,11]. Polysomnography, incorporating one or several electroencephalography (EEG) traces, is the gold standard for documentation of sleep and sleep stages in dogs, but this method has yet to be implemented in routine veterinary practice. Recently, the techniques for polysomnographic examinations in dogs were further developed [5,6,7,8]. Challenges are the sophisticated equipment; furthermore, PSG does not permit long-term recordings over days or even weeks, as actigraphy, the examination is time-consuming and interpretation requires considerable experience [5,6,7,8,12].

The written documentation and the accelerometer counts matched very well, but the study design without synchronized video data did not permit formal statistical analysis for inter-observer reliability, or to validate subjective behavioral classifications. Additional synchronized video recordings would have been advantageous to verify the observations, but the authors and owners opted against a technically elaborate camera set-up in the dogs’ home environment to avoid any disruption of the dogs’ daily routines and associated resting times. In this study, the dogs were able to choose their preferred resting zones (dog bed, sofa, etc.), and their sleep and rest behavior was not affected by an unfamiliar environment [9]. The individual sleep and resting periods of the dogs lasted only a few hours. Thus, it appeared unlikely that the observer’s ability to focus and concentrate was impaired. Furthermore, observers were veterinarians and veterinary technicians, and the quality checks, which were based on minute-to-minute comparisons with the accelerometer counts, indicated reliable observations. Each device was placed in a metal protective cover to protect it from damage, e.g., by getting chewed on or in contact with water, and mounted on a neck collar. No additional collar or leash was attached to avoid a possible influence caused by a “wrong movement” of the device [69]. This ensured that the devices were protected and could stay in place for future long-term recordings [26]. Depending on the chosen epoch, life, and battery life, the recording periods could last up to 200 days [70]. This would allow reliable long-term recordings over several weeks and months without internet access [70]. Limitations were that the performance data of the study accounted only for when dogs were monitored during their sleeping and resting periods, for example, in the afternoon or at night. The positive and negative predictive values could vary if the proportion of time spent resting or sleeping is different from this experiment. However, likelihood ratios support the use of the cut-off in different situations. Other limitations were that only healthy dogs and large breed dogs participated, thus findings may not generalize to smaller and more active breeds. Furthermore, mild to moderate sleep-related movements were not reliably detected which may affect utility in disorders with these signs. Lastly, the accelerometer is only able to detect quiescence and document rest–activity patterns. The accelerometer cannot detect sleep architecture, and the binary cut-off cannot detect when the dogs are awake and motionless (e.g., alert but still behavior).

In summary, the data obtained in this pilot study suggest that Actical^®^ actigraphy with a simple binary cut-off can provide a close estimate of the time that a dog spends sleeping and resting during its usual sleeping and resting periods. This mirrors the use of actigraphy for sleep monitoring in humans. Mild and moderate movements during sleeping and resting, without any change in the position of the head, neck, or trunk, are not documented by the accelerometer and, therefore, are not rated as interruptions of the sleeping and resting period. The data suggest the use of Actical^®^ actigraphy as a feasible tool to supplement caretakers’ reports. However, methodological limitations related to the sample size reduce the power and interpretability of the results. Further validation of this concept in larger cohorts with long-term monitoring is warranted, considering the significant practical implications for clinical trials and long-term monitoring. Potential indications for sleep and rest monitoring in dogs are sleep-related breathing disorders such as brachycephalic obstructive airway syndrome, restlessness during the night because of chronic pain from osteoarthritis/dental disease or disturbed sleep–wake cycles in elderly dogs with canine cognitive dysfunction syndrome [36,38,56,57,71].

## 5. Conclusions

Actical^®^ actigraphy was identified as a simple and objective method to document sleeping and resting behavior in dogs in this pilot study. The neck-mounted accelerometer could provide objective data on the duration of sleeping and resting periods in dogs. It offers compelling perspectives to obtain objective longitudinal data on sleeping and resting behavior and can complement the subjective reports of owners and caretakers. This appears especially important in clinical trials of neurologic diseases that may influence sleeping and resting behavior [58,71,72,73]. Further validation of this concept in larger cohorts with long-term monitoring is warranted to establish actigraphy as an objective monitoring tool and as a supplement for owner-reported outcome measures.

## Figures and Tables

**Table 1:** Study dogs and summary of observed sleep and rest periods.

Dog No.	Breed	Age (Months)	Sex ^#^	Total Observation Time (min *)	Valid Observation Time (min *)	Number of Observed Sleep and Rest Periods (n)	Mean Duration of Sleep and Rest Periods (min *)	Observer ^§^	Movements Reminiscent of REM ^&^ Sleep
1	Weimaraner	70	fs	305	301	3	102	vet 2	no
2	Labrador Retriever	28	m	479	468	9	53	vet 3	yes
3	Rhodesian Ridgeback	34	mn	202	202	5	40	vet 1	no
4	German Shepherd	79	fs	406	405	2	203	vet 4	no
5	Labrador Retriever	26	fs	173	173	3	58	vet 1	yes
6	Mixed Breed	206	fs	192	192	1	192	vet 5	no
7	Irish Setter	119	fs	255	255	3	85	vet 1	yes
8	Bernese Mountain Dog	46	fs	243	243	4	61	vet 1	yes
9	Dalmatian	17	f	182	182	1	182	vet 1	yes
10	Labrador Retriever	32	f	196	196	1	196	vet 1	yes
all dogs		40 (median)		2633 (total)	2617 (total)	3 (median)	94 (median)		

^#^ f, female; fs, female-spayed; m, male; mn, male-neutered; * min, minutes; ^§^ vet 1, first author (SSK); vet 2–5, observed by a veterinarian or veterinary technician who was also the pet owner; ^&^ REM, rapid eye movement.

**Table 2:** Movement categories.

M0	no movement	dog lying motionless in individual sleeping position with head resting on the ground; without any visible movement of the head, trunk, or limbs; eyelid movements could occur
M1	mild movement	dog lying in individual sleeping position, usually with head resting on the ground; only mild movements in this position, e.g., twitching of the nose, paws, or lips, nodding of the head
M2	moderate movement	dog lying in individual sleeping position, usually with head resting on the ground; only moderate movements in this position, e.g., changing position of the head, stretching the limbs; few (<5) scratching movements of the limbs could occur without a change in body position
M3	strong movement	dog changes body position, e.g., lying down, sitting or standing up
M4	awake, purposeful activities	dog walks around and interacts with the environment, e.g., playing, eating, drinking, other

**Table 3:** Performance of the accelerometer cut-off for documentation of movements and behavioral state.

	Observation (min)	cpm (Range)	0 cpm (min)	≥1 cpm (min)	Accelerometer Documents Movements	Accelerometer Documents Behavioral States
apparent sleep	1978	0–81	1942	36	-	97.7% (1942/1978)
movements						
no	1628	0–47	1618	10	0.6% (10/1628)	99.4% (1618/1628)
mild	292	0–81	285	7	2.4% (7/292)	97.6% (285/292)
moderate	58	0–59	39	19	32.8% (19/58)	67.2% (39/58)
rest	385	0–354	280	105	-	72.7% (280/385)
movements						
no	124	0	124	0	0%	100% (124/124)
mild	83	0–35	79	4	4.8% (4/83)	95.2% (79/83)
moderate	178	0–354	77	101	56.7% (101/178)	43.3% (77/178)
sleep and rest combined	2363	0–354	2222	141	-	94.0% (2222/2363)
movements						
no	1752	0–47	1742	10	0.6% (10/1752)	99.4% (1742/1752)
mild	375	0–81	364	11	2.9% (11/375)	97.1% (364/375)
moderate	23	0–354	116	120	50.8% (120/236)	49.2% (116/236)
awake and alert	254	12–7140	10	244	-	96.1% (244/254)
movements						-
no	10	0	10	0	0% (0/10)	0.0% (0/10)
strong	49	12–710	0	49	100% (49/49)	100.0% (49/49)
purposeful activities	195	12–7140	0	195	100% (195/195)	100.0% (105/195)
overall accuracy *	2617					94.2% (2466/2617)

* When used for monitoring combined sleeping and resting periods; cpm = accelerometer counts per minute.

**Table 4:** Performance of the Actical^®^ accelerometer cut-off for monitoring sleeping and resting periods (*p* < 0.001 for both behavioral states, McNemar test).

Behavioral State	Results	95%-Confidence Interval
monitoring of combined sleeping and resting periods		
sensitivity	0.940	0.930–0.950
specificity	0.961	0.929–0.981
positive likelihood ratio *	23.884	13.009–43.850
negative likelihood ratio *	0.062	0.053–0.073
area under the curve	0.950	
accuracy * (correctly classified proportion)	0.942	0.933–0.951
differentiation between sleeping and resting		
sensitivity	0.982	0.975–0.987
specificity	0.273	0.229–0.320
positive predictive value *	0.874	0.859–0.888
negative predictive value *	0.745	0.664–0.814
positive likelihood ratio	1.350	1.269–1.436
negative likelihood ratio	0.067	0.046–0.096
area under the curve	0.627	
accuracy * (correctly classified proportion)	0.866	0.852–0.880

* When used for monitoring of combined sleeping and resting periods.

## Data Availability

The data that supports the findings of this study are available within the article. The raw data are available from the corresponding author.
